# Crystal structure of bromido-*fac*-tricarbon­yl[5-(3,4,5-tri­meth­oxy­phen­yl)-3-(pyridin-2-yl)-1*H*-1,2,4-triazole-κ^2^ *N* ^2^,*N* ^3^]rhenium(I) methanol monosolvate

**DOI:** 10.1107/S2056989017003371

**Published:** 2017-03-10

**Authors:** Marharyta I. Kharlova, Kseniia O. Piletska, Kostiantyn V. Domasevitch, Alexander V. Shtemenko

**Affiliations:** a Department of Inorganic Chemistry, Ukrainian State University of Chemical Technology, Gagarin Ave. 8, Dnipro 49005, Ukraine; b Inorganic Chemistry Department, National Taras Shevchenko University of Kyiv, Volodymyrska Street 64/13, Kyiv 01601, Ukraine

**Keywords:** crystal structure, rhenium(I) carbonyl complex, 5-(3,4,5-tri­meth­oxy­phen­yl)-3-(pyridin-2-yl)-1*H*-1,2,4-triazole

## Abstract

In the title compound, [ReBr(C_16_H_16_N_4_O_3_)(CO)_3_]·CH_3_OH, the Re^I^ atom adopts a distorted octa­hedral coordination sphere with a facial arrangement of the three carbonyl ligands. Two N atoms of the chelating 5-(3,4,5-tri­meth­oxy­phen­yl)-3-(pyridin-2-yl)-1*H*-1,2,4-triazole ligand and two carbonyl ligands define the equatorial plane of the complex, with the third carbonyl ligand and the bromide ligand in axial positions. Conventional hydrogen bonds including the methanol solvent mol­ecules assemble the complex mol­ecules through mutual N—H⋯O—H⋯Br links [N⋯O = 2.703 (3) Å and O⋯Br = 3.255 (2) Å] into centrosymmetric dimers, whereas weaker C—H⋯O and C—H⋯Br hydrogen bonds [C⋯O = 3.215 (3)–3.390 (4) Å and C⋯Br = 3.927 (3) Å] connect the dimers into double layers parallel to the (111) plane.

## Abstract 2

The Re^I^ atom in the title methanol solvate is coordinated octa­hedrally by two N atoms of the chelating organic ligand, one Br atom and three facially configured carbonyl ligands. Hydrogen bonds between the complex and methanol solvent mol­ecules lead to a layered arrangement in the structure.

## Chemical context   

Rhenium(I) metal complexes have attracted attention because of their chemical characteristics exhibiting increased potentials for biochemical applications (Fernández-Moreira *et al.*, 2010 ▸; Lo *et al.*, 2012 ▸). Rhenium tricarbonyl complexes with the general formula *fac*-[Re(CO)_3_(N^N)] (where N^N is an *N*,*N*′-chelating ligand) are kinetically stable and have luminescence properties with long life times (Kowalski *et al.*, 2015 ▸; Guo *et al.*, 1997 ▸), high photostability (Lo, 2015 ▸) and large Stokes shifts (Lo, 2015 ▸; Stephenson *et al.*, 2004 ▸), which makes these compounds ideal candidates for either *in vitro* or *in vivo* visualization of biological processes (Shen *et al.*, 2001 ▸; Thorp-Greenwood, 2012 ▸).

**Figure 1:**
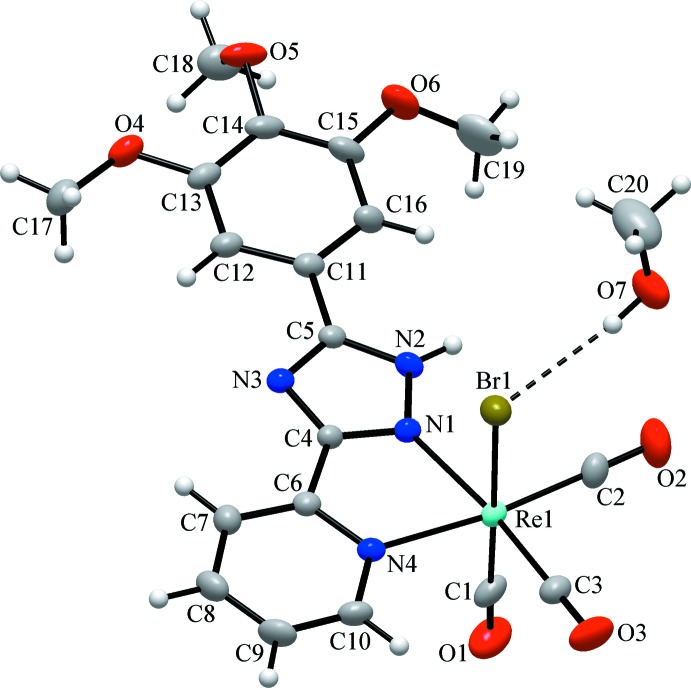
The structures of the mol­ecular entities in the solvated title complex. Displacement ellipsoids are drawn at the 40% probability level and the dashed line indicates hydrogen bonding involving the methanol solvent mol­ecule.

Triazole derivatives are an inter­esting type of ligand. 1,2,4-Triazoles have biological relevance since they show anti­viral (Abdullah *et al.*, 2012 ▸), anti­bacterial (Varvarason *et al.*, 2000 ▸; Jassim *et al.*, 2011 ▸), anti­fungal (Luo *et al.*, 2009 ▸), anti­cancer (Sztanke *et al.*, 2008 ▸) and anti­tuberculous (Mandal *et al.*, 2010 ▸) activities. Moreover, metal complexes containing triazole derivatives have inter­esting photophysical and photochemical properties (Piletska *et al.*, 2015 ▸; Chen *et al.*, 2013 ▸), and this class of complexes, apart from their biological activity (Chohan & Hanif, 2010 ▸), is used for fluorescent probing in addition to their potential use in radio-imaging. Introduction of substituents in the triazole derivatives affects the σ-donor and π-acceptor properties (Van Diemen *et al.*, 1991 ▸), and consequently affects the photophysical properties of an organometallic compounds in which they are incorporated. In this context, we report here the synthesis and crystal structure analysis of a novel Re^I^ complex, *i.e.* [ReBr(C_16_H_16_N_4_O_3_)(CO)_3_]·CH_3_OH (Fig. 1 ▸), which contains the triazole ligand 5-(3,4,5-tri­meth­oxy­phen­yl)-3-(pyridin-2-yl)-1*H*-1,2,4-triazole.
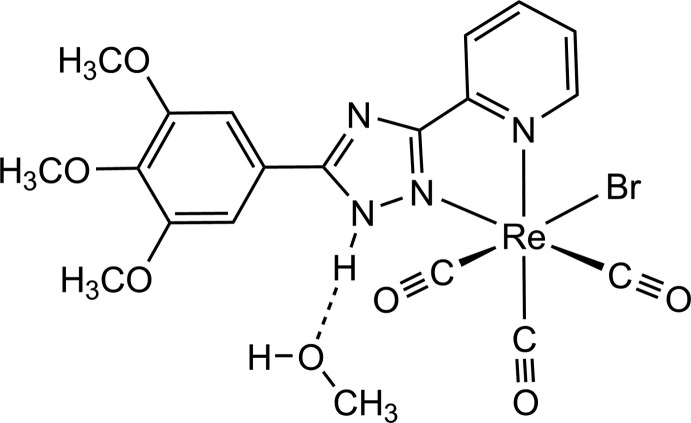


## Structural commentary   

The three carbonyl ligands bonded to the Re^I^ atom are arranged in a *fac* configuration. The distances of atoms C1, C2 and C3 to the Re^I^ atom are 1.902 (4), 1.910 (2) and 1.907 (2) Å, respectively, and the Re—N bond lengths involving the chelating organic ligand are 2.151 (2) and 2.205 (2) Å. The two N atoms and two carbonyl C atoms define the equatorial plane, while the octa­hedral coordination sphere is completed by the third carbonyl C atom and the Br atom [Re—Br = 2.6222 (3) Å] in axial positions. The CO ligands are almost linearly coordinated, with O—C—Re bond angles of 178.2 (3), 177.8 (3) and 177.8 (3)°. The C—Re—C bond angles between carbonyl C atoms are 90.6 (1), 90.2 (1) and 88.7 (1)°, close to ideal values, whereas the *cis* equatorial bite angle of the chelating ligand (N1—Re1—N4) is 73.42 (8)°.

## Supra­molecular features   

**Figure 2:**
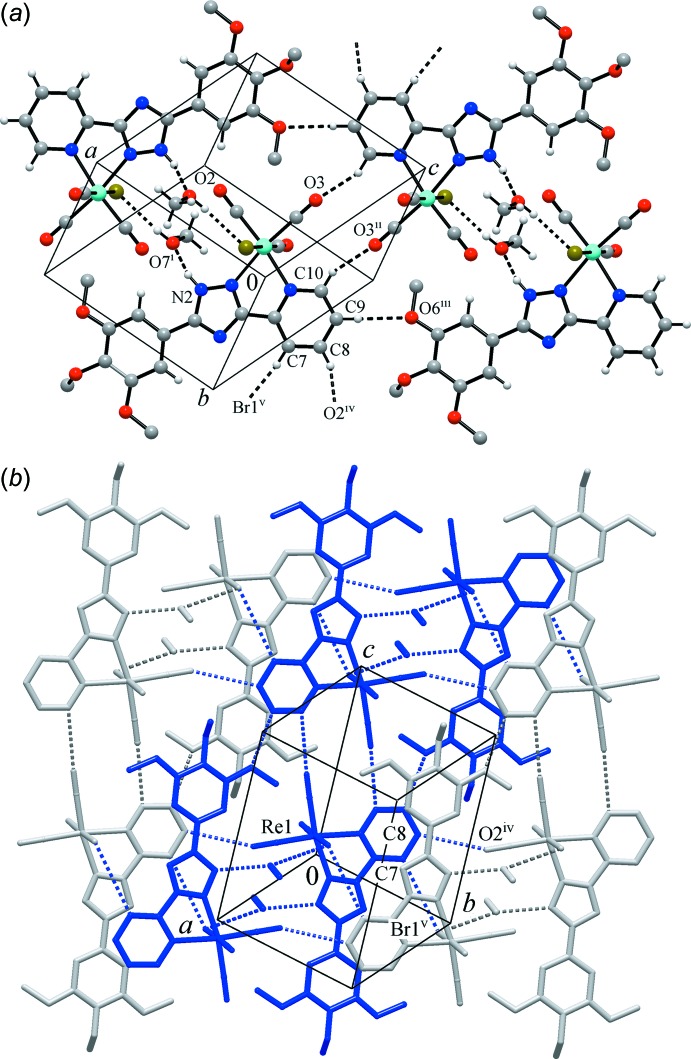
(*a*) Part of the crystal structure of the title complex, showing dimers formed by conventional hydrogen bonding involving the methanol solvent mol­ecules and weak C—H⋯O inter­actions providing inter­connection of the dimers into chains. (*b*) A partial view of the double layer in a projection approximately on the (111) plane; individual chains are marked in blue and grey and the dotted lines indicate hydrogen bonding within a layer. [Symmetry codes: (i) 1 − *x*, −*y*, −*z*; (ii) −*x*, −*y*, 1 − *z*; (iii) −1 + *x*, *y*, 1 + *z*; (iv) −1 + *x*, 1 + *y*, *z*; (v) −*x*, 1 − *y*, −*z*.]

In the crystal, the packing of the mol­ecules is influenced by a set of weak inter­actions, including conventional hydrogen bonding with common NH and OH donor groups and weaker hydrogen bonds formed by CH groups (Table 1 ▸). Two pairs of relatively short hydrogen bonds (O7—H⋯Br1 and N2—H⋯O7), both involving the methanol solvent mol­ecules, assemble the complex mol­ecules into centrosymmetric dimers (Fig. 2 ▸). As may be compared with the closely related complex [ReBr(*L*)(CO)_3_] [*L* = 5-phenyl-3-(pyridin-2-yl)-1*H*-1,2,4-triazole; Piletska *et al.*, 2014 ▸], a key prerequisite for the formation of dimers is the presence of acidic NH functions and sterically accessible Br sites. In the latter, they afford two mutual N—H⋯Br hydrogen bonds, whereas in the present case, these links appear to be extended by the inclusion of methanol, resulting in an N—H⋯O(Me)—H⋯Br motif.

**Figure 3:**
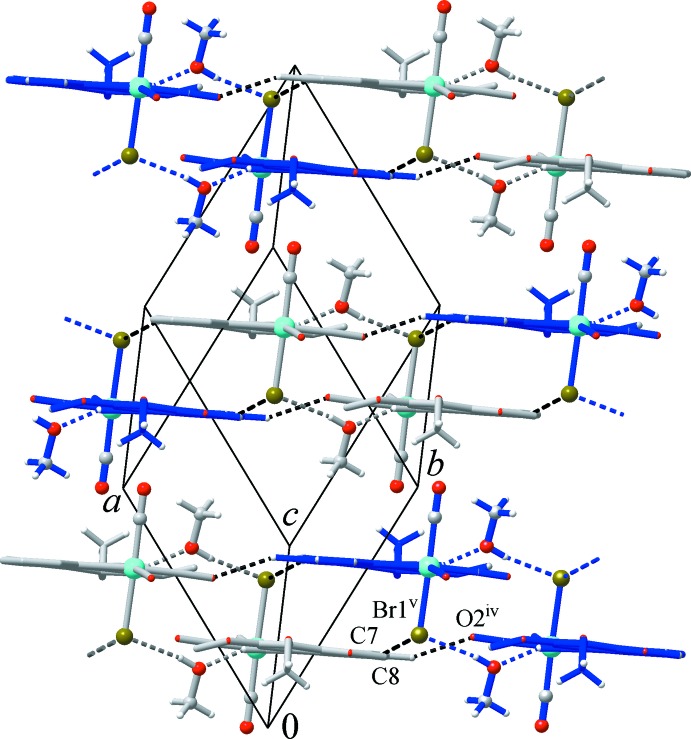
Packing of successive double layers, which are turned towards one another by the methyl and carbonyl groups (the view is along the direction of the hydrogen-bonded chains indicated with blue and grey bonds). [Symmetry codes: (iv) −1 + *x*, 1 + *y*, *z*; (v) −*x*, 1 − *y*, −*z*.]

Each of the four pyridine CH groups functions as a donor of weak hydrogen bonds (Fig. 2 ▸). These groups establish hydrogen bonds to two carbonyl O atoms (C8⋯O2^iv^ and C10⋯O3^ii^), a meth­oxy O atom (C9⋯O6^iii^) and a very weak bond with bromine as acceptor (C7⋯Br^v^) (for symmetry codes, see Table 1 ▸). These distal yet directional inter­actions (the hydrogen-bonding angles are in the range 142–165°; Table 1 ▸) unite the above dimers into flat double layers, which extend parallel to the (111) plane. Within a layer, the pyridine and triazole moieties of adjacent mol­ecules are actually parallel, with shortest contacts of C7⋯N3^v^ = 3.430 (4) Å [symmetry code: (v) −*x*, 1 − *y*, −*z*]. However, this situation is unlikely to be a consequence of slipped π–π inter­actions, since the corresponding slippage angle exceeds 56° and the inter­centroid distance is as long as *Cg*(C6–C10/N4)⋯*Cg*(C4/C5/N1–N3)^v^ = 4.090 (3) Å [for the lack of an overlap between heteroaromatic planes, see Fig. 2 ▸, part (B)]. At the same time, successive double layers are turned towards one another by methyl groups of the tri­meth­oxy­phenyl and methanol entities (Fig. 3 ▸). Thus, the inter­layer inter­actions are very weak and the only remarkable contact is found between two inversion-related carbonyl groups [O1⋯C1^vi^ = 3.295 (3) Å and O1⋯*Cg*(C1=O1)^vi^ = 3.226 (3) Å; symmetry code (vi) −*x*, −*y*, −*z*]. Although such weak inter­actions are characteristic of related metal–carbonyl structures (Sparkes *et al.*, 2006 ▸), in the present case, their significance is relatively minor.

## Synthesis and crystallization   

Penta­carbonyl­rhenium(I) bromide (0.15 g, 0.369 mmol) was mixed with 5-(3,4,5-tri­meth­oxy­phen­yl)-3-(pyridin-2-yl)-1*H*-1,2,4-triazole (0.138 g, 0.442 mmol) in benzene (30 ml). The mixture was refluxed for 4 h under a stream of argon and then allowed to cool to room temperature. The yellow product was collected by suction filtration, washed with hexane and dried (yield 0.138 g, 77%). Crystals suitable for X-ray diffraction were obtained by slow diffusion of hexane into a methanol–di­chloro­methane solution of the complex. IR (KBr, cm^−1^): ν_as_(CO) 2028 (*s*), ν_s_(CO) 1894 (*s*). ^1^H NMR (400 MHz, *d* ^6^-DMSO): δ 9.02 (*d*, 1H, CH=N, Py), 8.39 (*d*, 1H, CH=C, Py), 8.35 (*dd*, 1H, CH, Py), 7.75 (*dd*, 1H, CH, Py), 7.44 [*s*, 2H, 2 CH, Ph(OCH_3_)_3_], 3.93 [*s*, 9H, 3 O-CH_3_, Ph(OCH_3_)_3_].

## Refinement   

Crystal data, data collection and structure refinement details are summarized in Table 2 ▸. C- and N-bound H atoms were positioned with idealized geometry and were refined with aryl C—H = 0.94 Å, methyl C—H = 0.97 Å and N—H = 0.87 Å, and with *U*_iso_(H) = 1.5*U*_eq_(C) for methyl H atoms and 1.2*U*_eq_(C,N) otherwise. The O-bound H atom of the methanol solvent mol­ecule was found from a difference map and was refined with O—H = 0.95 Å and *U*_iso_(H) = 1.5*U*_eq_(O).

## Supplementary Material

Crystal structure: contains datablock(s) I. DOI: 10.1107/S2056989017003371/wm5372sup1.cif

Structure factors: contains datablock(s) I. DOI: 10.1107/S2056989017003371/wm5372Isup2.hkl

CCDC reference: 1535292

Additional supporting information:  crystallographic information; 3D view; checkCIF report

## Figures and Tables

**Table 1:** Hydrogen-bond geometry (Å, °)

*D*—H⋯*A*	*D*—H	H⋯*A*	*D*⋯*A*	*D*—H⋯*A*
O7—H1*O*⋯Br1	0.85	2.41	3.255 (2)	172
N2—H1*N*⋯O7^i^	0.87	1.89	2.703 (3)	154
C7—H7⋯Br1^ii^	0.94	3.01	3.927 (3)	165
C8—H8⋯O2^iii^	0.94	2.52	3.390 (4)	153
C9—H9⋯O6^iv^	0.94	2.49	3.278 (3)	142
C10—H10⋯O3^v^	0.94	2.39	3.215 (3)	146

Symmetry codes: (i) 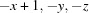; (ii) 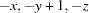; (iii) 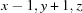; (iv) 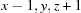; (v) 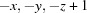.

**Table 2:** Experimental details

Crystal data	
Chemical formula	[ReBr(C_16_H_16_N_4_O_3_)(CO)_3_]·CH_4_O
*M*_r_	694.51
Crystal system, space group	Triclinic, *P* 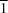
Temperature (K)	213
*a*, *b*, *c* (Å)	10.9569 (6), 11.0012 (6), 11.9738 (7)
α, β, γ (°)	69.073 (6), 75.593 (7), 61.409 (6)
*V* (Å^3^)	1178.37 (14)
*Z*	2
Radiation type	Mo *K*α
μ (mm^−1^)	6.90
Crystal size (mm)	0.21 × 0.18 × 0.15

Data collection	
Diffractometer	Stoe IPDS
Absorption correction	Numerical (*X-RED* and *X-SHAPE*; Stoe & Cie, 1999 ▸)
*T*_min_, *T*_max_	0.325, 0.424
No. of measured, independent and observed [*I* > 2σ(*I*)] reflections	22150, 5649, 4658
*R*_int_	0.054
(sin θ/λ)_max_ (Å^−1^)	0.660

Refinement	
*R*[*F* ^2^ > 2σ(*F* ^2^)], *wR*(*F* ^2^), *S*	0.019, 0.034, 0.84
No. of reflections	5649
No. of parameters	302
H-atom treatment	H-atom parameters constrained
Δρ_max_, Δρ_min_ (e Å^−3^)	0.77, −0.98

Computer programs: *IPDS* (Stoe & Cie, 2000 ▸), *SHELXS97* and *SHELXL97* (Sheldrick, 2008 ▸), *DIAMOND* (Brandenburg, 1999 ▸) and *WinGX* (Farrugia, 2012 ▸).

## supplementary crystallographic information

### Crystal data

**Table:**

[ReBr(C_16_H_16_N_4_O_3_)(CO)_3_]·CH_4_O	*Z* = 2
*M**_r_* = 694.51	*F*(000) = 668
Triclinic, *P*1	*D*_x_ = 1.957 Mg m^−^^3^
*a* = 10.9569 (6) Å	Mo *K*α radiation, λ = 0.71073 Å
*b* = 11.0012 (6) Å	Cell parameters from 8000 reflections
*c* = 11.9738 (7) Å	θ = 2.4–28.0°
α = 69.073 (6)°	µ = 6.90 mm^−^^1^
β = 75.593 (7)°	*T* = 213 K
γ = 61.409 (6)°	Prism, yellow
*V* = 1178.37 (14) Å^3^	0.21 × 0.18 × 0.15 mm

### Data collection

**Table:**

Stoe IPDS diffractometer	5649 independent reflections
Radiation source: fine-focus sealed tube	4658 reflections with *I* > 2σ(*I*)
Graphite monochromator	*R*_int_ = 0.054
φ oscillation scans	θ_max_ = 28.0°, θ_min_ = 2.4°
Absorption correction: numerical (*X-RED* and *X-SHAPE*; Stoe & Cie, 1999)	*h* = −14→14
*T*_min_ = 0.325, *T*_max_ = 0.424	*k* = −14→14
22150 measured reflections	*l* = −15→15

### Refinement

**Table:**

Refinement on *F*^2^	Primary atom site location: structure-invariant direct methods
Least-squares matrix: full	Secondary atom site location: difference Fourier map
*R*[*F*^2^ > 2σ(*F*^2^)] = 0.019	Hydrogen site location: inferred from neighbouring sites
*wR*(*F*^2^) = 0.034	H-atom parameters constrained
*S* = 0.84	*w* = 1/[σ^2^(*F*_o_^2^) + (0.007*P*)^2^] where *P* = (*F*_o_^2^ + 2*F*_c_^2^)/3
5649 reflections	(Δ/σ)_max_ = 0.002
302 parameters	Δρ_max_ = 0.77 e Å^−^^3^
0 restraints	Δρ_min_ = −0.98 e Å^−^^3^

### Special details

**Table:**

Geometry. All esds (except the esd in the dihedral angle between two l.s. planes) are estimated using the full covariance matrix. The cell esds are taken into account individually in the estimation of esds in distances, angles and torsion angles; correlations between esds in cell parameters are only used when they are defined by crystal symmetry. An approximate (isotropic) treatment of cell esds is used for estimating esds involving l.s. planes.
Refinement. Refinement of F^2^ against ALL reflections. The weighted R-factor wR and goodness of fit S are based on F^2^, conventional R-factors R are based on F, with F set to zero for negative F^2^. The threshold expression of F^2^ > 2sigma(F^2^) is used only for calculating R-factors(gt) etc. and is not relevant to the choice of reflections for refinement. R-factors based on F^2^ are statistically about twice as large as those based on F, and R- factors based on ALL data will be even larger.

### Fractional atomic coordinates and isotropic or equivalent isotropic displacement parameters (Å^2^)

**Table:**

	*x*	*y*	*z*	*U*_iso_*/*U*_eq_	
Re1	0.154206 (13)	0.050031 (12)	0.169575 (9)	0.02510 (3)	
Br1	0.25284 (3)	0.20300 (3)	0.21180 (2)	0.03551 (7)	
O1	0.0338 (3)	−0.1259 (3)	0.1269 (2)	0.0619 (7)	
O2	0.4513 (3)	−0.1785 (3)	0.1342 (2)	0.0647 (7)	
O3	0.1502 (3)	−0.1065 (3)	0.43714 (17)	0.0564 (6)	
O4	0.0599 (2)	0.6925 (2)	−0.58379 (15)	0.0424 (5)	
O5	0.3238 (2)	0.5465 (3)	−0.66521 (15)	0.0496 (6)	
O6	0.4851 (2)	0.2842 (3)	−0.53647 (19)	0.0588 (7)	
O7	0.5906 (2)	0.0402 (3)	0.1603 (2)	0.0607 (6)	
H1O	0.5022	0.0743	0.1740	0.091*	
N1	0.1308 (2)	0.1987 (2)	−0.00751 (16)	0.0251 (5)	
N2	0.2059 (2)	0.2076 (2)	−0.11623 (17)	0.0277 (5)	
H1N	0.2870	0.1422	−0.1341	0.042*	
N3	0.0134 (2)	0.4101 (2)	−0.13551 (17)	0.0276 (5)	
N4	−0.0517 (2)	0.2340 (2)	0.17886 (16)	0.0261 (5)	
C1	0.0776 (3)	−0.0582 (3)	0.1415 (2)	0.0372 (7)	
C2	0.3389 (3)	−0.0940 (3)	0.1465 (2)	0.0374 (7)	
C3	0.1518 (3)	−0.0502 (3)	0.3364 (2)	0.0337 (6)	
C4	0.0180 (3)	0.3209 (3)	−0.02390 (19)	0.0236 (5)	
C5	0.1328 (3)	0.3353 (3)	−0.1915 (2)	0.0258 (5)	
C6	−0.0881 (3)	0.3444 (3)	0.0779 (2)	0.0256 (5)	
C7	−0.2134 (3)	0.4647 (3)	0.0716 (2)	0.0330 (6)	
H7	−0.2349	0.5395	−0.0004	0.040*	
C8	−0.3074 (3)	0.4739 (3)	0.1731 (3)	0.0393 (7)	
H8	−0.3944	0.5547	0.1712	0.047*	
C9	−0.2715 (3)	0.3632 (3)	0.2763 (2)	0.0402 (7)	
H9	−0.3335	0.3675	0.3465	0.048*	
C10	−0.1442 (3)	0.2460 (3)	0.2766 (2)	0.0346 (6)	
H10	−0.1209	0.1709	0.3482	0.042*	
C11	0.1808 (3)	0.3865 (3)	−0.3176 (2)	0.0292 (6)	
C12	0.0921 (3)	0.5169 (3)	−0.3878 (2)	0.0311 (6)	
H12	0.0019	0.5697	−0.3557	0.037*	
C13	0.1380 (3)	0.5683 (3)	−0.5059 (2)	0.0335 (6)	
C14	0.2732 (3)	0.4897 (3)	−0.5527 (2)	0.0374 (7)	
C15	0.3585 (3)	0.3574 (3)	−0.4825 (2)	0.0392 (7)	
C16	0.3131 (3)	0.3051 (3)	−0.3641 (2)	0.0363 (7)	
H16	0.3713	0.2156	−0.3160	0.044*	
C17	−0.0765 (4)	0.7778 (3)	−0.5387 (3)	0.0465 (8)	
H17A	−0.1313	0.7229	−0.5112	0.070*	
H17B	−0.1212	0.8642	−0.6019	0.070*	
H17C	−0.0701	0.8046	−0.4721	0.070*	
C18	0.2920 (4)	0.5205 (4)	−0.7592 (3)	0.0544 (9)	
H18A	0.3399	0.4181	−0.7525	0.082*	
H18B	0.3221	0.5733	−0.8358	0.082*	
H18C	0.1920	0.5521	−0.7538	0.082*	
C19	0.5582 (4)	0.1362 (5)	−0.4788 (4)	0.0857 (14)	
H19A	0.5877	0.1254	−0.4045	0.128*	
H19B	0.6395	0.0928	−0.5309	0.128*	
H19C	0.4977	0.0888	−0.4613	0.128*	
C20	0.6343 (5)	0.1423 (5)	0.1534 (5)	0.0861 (14)	
H20A	0.6044	0.1685	0.2282	0.129*	
H20B	0.5936	0.2272	0.0878	0.129*	
H20C	0.7353	0.1024	0.1395	0.129*	

### Atomic displacement parameters (Å^2^)

**Table:**

	*U*^11^	*U*^22^	*U*^33^	*U*^12^	*U*^13^	*U*^23^
Re1	0.02840 (6)	0.02390 (6)	0.02089 (5)	−0.01144 (4)	−0.00314 (3)	−0.00318 (3)
Br1	0.03337 (16)	0.03401 (17)	0.04178 (14)	−0.01567 (13)	−0.00360 (12)	−0.01181 (12)
O1	0.088 (2)	0.0571 (16)	0.0582 (14)	−0.0442 (15)	−0.0299 (13)	−0.0010 (12)
O2	0.0457 (15)	0.0503 (16)	0.0727 (16)	0.0050 (13)	−0.0118 (12)	−0.0217 (12)
O3	0.0822 (18)	0.0659 (16)	0.0272 (10)	−0.0472 (15)	−0.0099 (10)	0.0052 (10)
O4	0.0561 (14)	0.0372 (12)	0.0284 (9)	−0.0229 (11)	−0.0026 (9)	−0.0001 (8)
O5	0.0627 (15)	0.0786 (16)	0.0239 (9)	−0.0539 (14)	0.0066 (9)	−0.0060 (9)
O6	0.0325 (12)	0.0807 (19)	0.0418 (11)	−0.0187 (13)	0.0098 (9)	−0.0108 (11)
O7	0.0342 (13)	0.0493 (15)	0.0909 (17)	−0.0084 (12)	0.0067 (12)	−0.0335 (13)
N1	0.0260 (12)	0.0283 (12)	0.0190 (9)	−0.0126 (10)	0.0005 (8)	−0.0049 (8)
N2	0.0231 (11)	0.0347 (13)	0.0223 (10)	−0.0118 (10)	0.0026 (8)	−0.0090 (9)
N3	0.0268 (12)	0.0283 (12)	0.0256 (10)	−0.0136 (10)	0.0001 (9)	−0.0045 (9)
N4	0.0261 (12)	0.0302 (12)	0.0223 (9)	−0.0149 (10)	0.0005 (8)	−0.0057 (8)
C1	0.055 (2)	0.0295 (16)	0.0275 (13)	−0.0215 (15)	−0.0095 (12)	0.0000 (11)
C2	0.0396 (18)	0.0311 (17)	0.0327 (14)	−0.0084 (15)	−0.0097 (12)	−0.0050 (12)
C3	0.0403 (17)	0.0340 (16)	0.0288 (13)	−0.0204 (14)	−0.0052 (11)	−0.0036 (11)
C4	0.0228 (13)	0.0272 (14)	0.0210 (10)	−0.0123 (11)	−0.0026 (9)	−0.0042 (9)
C5	0.0245 (13)	0.0302 (15)	0.0245 (11)	−0.0156 (12)	−0.0021 (10)	−0.0042 (10)
C6	0.0267 (14)	0.0281 (14)	0.0252 (11)	−0.0151 (12)	−0.0013 (10)	−0.0071 (10)
C7	0.0294 (15)	0.0302 (16)	0.0363 (13)	−0.0111 (13)	−0.0043 (11)	−0.0075 (11)
C8	0.0273 (15)	0.0377 (18)	0.0515 (16)	−0.0126 (14)	0.0053 (13)	−0.0190 (14)
C9	0.0362 (17)	0.049 (2)	0.0382 (14)	−0.0234 (16)	0.0148 (12)	−0.0212 (14)
C10	0.0394 (17)	0.0419 (18)	0.0258 (12)	−0.0254 (15)	0.0047 (11)	−0.0070 (11)
C11	0.0306 (15)	0.0369 (16)	0.0239 (11)	−0.0202 (13)	−0.0006 (10)	−0.0062 (10)
C12	0.0366 (16)	0.0359 (16)	0.0246 (11)	−0.0220 (13)	−0.0003 (11)	−0.0054 (11)
C13	0.0464 (18)	0.0344 (16)	0.0251 (12)	−0.0260 (15)	−0.0045 (12)	−0.0012 (11)
C14	0.0446 (18)	0.056 (2)	0.0238 (12)	−0.0368 (16)	0.0029 (12)	−0.0070 (12)
C15	0.0284 (15)	0.062 (2)	0.0294 (13)	−0.0249 (15)	0.0052 (11)	−0.0125 (13)
C16	0.0296 (15)	0.0466 (18)	0.0267 (12)	−0.0179 (14)	−0.0026 (11)	−0.0015 (12)
C17	0.053 (2)	0.0380 (19)	0.0382 (15)	−0.0163 (16)	−0.0077 (14)	−0.0012 (13)
C18	0.064 (2)	0.075 (3)	0.0333 (15)	−0.039 (2)	0.0008 (15)	−0.0156 (16)
C19	0.045 (2)	0.098 (4)	0.087 (3)	−0.021 (2)	0.031 (2)	−0.036 (3)
C20	0.060 (3)	0.086 (3)	0.134 (4)	−0.029 (3)	−0.005 (3)	−0.062 (3)

### Geometric parameters (Å, º)

**Table:**

Re1—C1	1.902 (3)	C7—C8	1.383 (4)
Re1—C3	1.907 (3)	C7—H7	0.9400
Re1—C2	1.910 (3)	C8—C9	1.369 (4)
Re1—N1	2.1515 (18)	C8—H8	0.9400
Re1—N4	2.205 (2)	C9—C10	1.373 (4)
Re1—Br1	2.6222 (3)	C9—H9	0.9400
O1—C1	1.137 (3)	C10—H10	0.9400
O2—C2	1.146 (4)	C11—C12	1.385 (4)
O3—C3	1.142 (3)	C11—C16	1.388 (4)
O4—C13	1.355 (3)	C12—C13	1.386 (3)
O4—C17	1.423 (4)	C12—H12	0.9400
O5—C14	1.368 (3)	C13—C14	1.401 (4)
O5—C18	1.410 (4)	C14—C15	1.383 (4)
O6—C15	1.359 (4)	C15—C16	1.389 (4)
O6—C19	1.412 (5)	C16—H16	0.9400
O7—C20	1.388 (5)	C17—H17A	0.9700
O7—H1O	0.8500	C17—H17B	0.9700
N1—C4	1.310 (3)	C17—H17C	0.9700
N1—N2	1.354 (3)	C18—H18A	0.9700
N2—C5	1.344 (3)	C18—H18B	0.9700
N2—H1N	0.8700	C18—H18C	0.9700
N3—C5	1.329 (3)	C19—H19A	0.9700
N3—C4	1.342 (3)	C19—H19B	0.9700
N4—C10	1.344 (3)	C19—H19C	0.9700
N4—C6	1.350 (3)	C20—H20A	0.9700
C4—C6	1.462 (3)	C20—H20B	0.9700
C5—C11	1.468 (3)	C20—H20C	0.9700
C6—C7	1.372 (4)		
			
C1—Re1—C3	90.15 (11)	C8—C9—H9	120.2
C1—Re1—C2	90.62 (13)	C10—C9—H9	120.2
C3—Re1—C2	88.74 (12)	N4—C10—C9	122.8 (2)
C1—Re1—N1	95.06 (9)	N4—C10—H10	118.6
C3—Re1—N1	169.20 (10)	C9—C10—H10	118.6
C2—Re1—N1	100.64 (10)	C12—C11—C16	121.3 (2)
C1—Re1—N4	92.83 (11)	C12—C11—C5	118.7 (2)
C3—Re1—N4	96.92 (10)	C16—C11—C5	120.0 (2)
C2—Re1—N4	173.36 (10)	C11—C12—C13	119.2 (3)
N1—Re1—N4	73.41 (7)	C11—C12—H12	120.4
C1—Re1—Br1	178.32 (9)	C13—C12—H12	120.4
C3—Re1—Br1	89.23 (8)	O4—C13—C12	124.5 (3)
C2—Re1—Br1	90.92 (9)	O4—C13—C14	115.4 (2)
N1—Re1—Br1	85.31 (6)	C12—C13—C14	120.1 (3)
N4—Re1—Br1	85.70 (6)	O5—C14—C15	119.8 (3)
C13—O4—C17	117.1 (2)	O5—C14—C13	120.3 (3)
C14—O5—C18	114.9 (2)	C15—C14—C13	119.9 (2)
C15—O6—C19	117.1 (2)	O6—C15—C14	116.3 (2)
C20—O7—H1O	109.6	O6—C15—C16	123.4 (3)
C4—N1—N2	104.04 (18)	C14—C15—C16	120.3 (3)
C4—N1—Re1	118.20 (15)	C11—C16—C15	119.2 (3)
N2—N1—Re1	137.71 (17)	C11—C16—H16	120.4
C5—N2—N1	107.9 (2)	C15—C16—H16	120.4
C5—N2—H1N	126.1	O4—C17—H17A	109.5
N1—N2—H1N	126.1	O4—C17—H17B	109.5
C5—N3—C4	102.6 (2)	H17A—C17—H17B	109.5
C10—N4—C6	117.0 (2)	O4—C17—H17C	109.5
C10—N4—Re1	125.62 (18)	H17A—C17—H17C	109.5
C6—N4—Re1	117.35 (15)	H17B—C17—H17C	109.5
O1—C1—Re1	178.2 (3)	O5—C18—H18A	109.5
O2—C2—Re1	177.9 (3)	O5—C18—H18B	109.5
O3—C3—Re1	177.7 (3)	H18A—C18—H18B	109.5
N1—C4—N3	114.7 (2)	O5—C18—H18C	109.5
N1—C4—C6	117.8 (2)	H18A—C18—H18C	109.5
N3—C4—C6	127.5 (2)	H18B—C18—H18C	109.5
N3—C5—N2	110.8 (2)	O6—C19—H19A	109.5
N3—C5—C11	124.8 (2)	O6—C19—H19B	109.5
N2—C5—C11	124.4 (2)	H19A—C19—H19B	109.5
N4—C6—C7	123.1 (2)	O6—C19—H19C	109.5
N4—C6—C4	113.1 (2)	H19A—C19—H19C	109.5
C7—C6—C4	123.8 (2)	H19B—C19—H19C	109.5
C6—C7—C8	118.8 (3)	O7—C20—H20A	109.5
C6—C7—H7	120.6	O7—C20—H20B	109.5
C8—C7—H7	120.6	H20A—C20—H20B	109.5
C9—C8—C7	118.7 (3)	O7—C20—H20C	109.5
C9—C8—H8	120.6	H20A—C20—H20C	109.5
C7—C8—H8	120.6	H20B—C20—H20C	109.5
C8—C9—C10	119.5 (2)		
			
C1—Re1—N1—C4	94.2 (2)	N1—C4—C6—C7	−177.2 (3)
C3—Re1—N1—C4	−24.3 (7)	N3—C4—C6—C7	1.7 (4)
C2—Re1—N1—C4	−174.2 (2)	N4—C6—C7—C8	−0.1 (4)
N4—Re1—N1—C4	2.79 (18)	C4—C6—C7—C8	178.5 (2)
Br1—Re1—N1—C4	−84.12 (18)	C6—C7—C8—C9	0.6 (4)
C1—Re1—N1—N2	−88.8 (3)	C7—C8—C9—C10	−0.4 (4)
C3—Re1—N1—N2	152.7 (5)	C6—N4—C10—C9	0.6 (4)
C2—Re1—N1—N2	2.8 (3)	Re1—N4—C10—C9	−179.5 (2)
N4—Re1—N1—N2	179.8 (3)	C8—C9—C10—N4	−0.2 (5)
Br1—Re1—N1—N2	92.9 (2)	N3—C5—C11—C12	−5.7 (4)
C4—N1—N2—C5	−0.1 (3)	N2—C5—C11—C12	176.3 (2)
Re1—N1—N2—C5	−177.32 (19)	N3—C5—C11—C16	174.0 (3)
C1—Re1—N4—C10	83.7 (2)	N2—C5—C11—C16	−4.0 (4)
C3—Re1—N4—C10	−6.7 (2)	C16—C11—C12—C13	−1.6 (4)
N1—Re1—N4—C10	178.2 (2)	C5—C11—C12—C13	178.1 (2)
Br1—Re1—N4—C10	−95.4 (2)	C17—O4—C13—C12	2.6 (4)
C1—Re1—N4—C6	−96.34 (19)	C17—O4—C13—C14	−178.0 (3)
C3—Re1—N4—C6	173.17 (19)	C11—C12—C13—O4	178.6 (3)
N1—Re1—N4—C6	−1.90 (18)	C11—C12—C13—C14	−0.8 (4)
Br1—Re1—N4—C6	84.47 (18)	C18—O5—C14—C15	96.3 (3)
N2—N1—C4—N3	−0.3 (3)	C18—O5—C14—C13	−87.1 (4)
Re1—N1—C4—N3	177.60 (16)	O4—C13—C14—O5	7.0 (4)
N2—N1—C4—C6	178.8 (2)	C12—C13—C14—O5	−173.6 (2)
Re1—N1—C4—C6	−3.3 (3)	O4—C13—C14—C15	−176.4 (3)
C5—N3—C4—N1	0.5 (3)	C12—C13—C14—C15	3.0 (4)
C5—N3—C4—C6	−178.4 (2)	C19—O6—C15—C14	−164.1 (3)
C4—N3—C5—N2	−0.6 (3)	C19—O6—C15—C16	15.2 (5)
C4—N3—C5—C11	−178.8 (2)	O5—C14—C15—O6	−6.9 (4)
N1—N2—C5—N3	0.4 (3)	C13—C14—C15—O6	176.4 (3)
N1—N2—C5—C11	178.7 (2)	O5—C14—C15—C16	173.7 (3)
C10—N4—C6—C7	−0.4 (4)	C13—C14—C15—C16	−2.9 (4)
Re1—N4—C6—C7	179.7 (2)	C12—C11—C16—C15	1.7 (4)
C10—N4—C6—C4	−179.2 (2)	C5—C11—C16—C15	−178.0 (3)
Re1—N4—C6—C4	0.9 (3)	O6—C15—C16—C11	−178.7 (3)
N1—C4—C6—N4	1.6 (3)	C14—C15—C16—C11	0.6 (4)
N3—C4—C6—N4	−179.5 (2)		

### Hydrogen-bond geometry (Å, º)

**Table:**

*D*—H···*A*	*D*—H	H···*A*	*D*···*A*	*D*—H···*A*
O7—H1*O*···Br1	0.85	2.41	3.255 (2)	172
N2—H1*N*···O7^i^	0.87	1.89	2.703 (3)	154
C7—H7···Br1^ii^	0.94	3.01	3.927 (3)	165
C8—H8···O2^iii^	0.94	2.52	3.390 (4)	153
C9—H9···O6^iv^	0.94	2.49	3.278 (3)	142
C10—H10···O3^v^	0.94	2.39	3.215 (3)	146

Symmetry codes: (i) −*x*+1, −*y*, −*z*; (ii) −*x*, −*y*+1, −*z*; (iii) *x*−1, *y*+1, *z*; (iv) *x*−1, *y*, *z*+1; (v) −*x*, −*y*, −*z*+1.
